# Soluble RAGE and atherosclerosis in youth with type 1 diabetes: a 5-year follow-up study

**DOI:** 10.1186/s12933-015-0292-2

**Published:** 2015-09-25

**Authors:** Martin Heier, Hanna Dis Margeirsdottir, Mario Gaarder, Knut Haakon Stensæth, Cathrine Brunborg, Peter Abusdal Torjesen, Ingebjørg Seljeflot, Kristian Folkvord Hanssen, Knut Dahl-Jørgensen

**Affiliations:** Pediatric Department, Oslo University Hospital, Oslo, Norway; Faculty of Medicine, University of Oslo, Oslo, Norway; Oslo Diabetes Research Centre, Oslo, Norway; Akershus University Hospital, Lørenskog, Norway; Department of Radiology and Nuclear Medicine, Oslo University Hospital, Oslo, Norway; Department of Biostatistics and Epidemiology, Oslo University Hospital, Oslo, Norway; Hormone Laboratory, Oslo University Hospital, Oslo, Norway; Department of Cardiology, Center for Clinical Heart Research, Oslo University Hospital, Oslo, Norway; Department of Endocrinology, Oslo University Hospital, Oslo, Norway

**Keywords:** Advanced glycation end products, Receptor for advanced glycation end products, sRAGE, esRAGE, Atherosclerosis, Type 1 diabetes, CRP

## Abstract

**Background:**

Advanced glycation end products (AGEs) play a role in the development of late complications and atherosclerosis in diabetes by engaging the receptor for advanced glycation end products, RAGE. Receptor binding leads to activation of the vascular endothelium and increased inflammation in the vessel wall. The soluble variants of the receptor, endogenous secretory RAGE (esRAGE) and the cleaved cell-surface part of RAGE, which together comprise soluble RAGE (sRAGE), are suggested to have a protective effect acting as decoys for RAGE. We aimed to test whether high levels of soluble variants of RAGE could be protective against atherosclerosis development.

**Methods:**

Participants in the prospective atherosclerosis and childhood diabetes study were examined at baseline (aged 8–18) and at follow-up after 5 years. Both sRAGE and esRAGE were measured by immunoassay in 299 patients with type 1 diabetes and 112 healthy controls at baseline and 241 patients and 128 controls at follow-up. The AGEs methylglyoxal-derived hydroimidazolone-1 (MG-H1) and carboxymethyllysine (CML) were measured by immunoassay. The surrogate markers of atherosclerosis assessed were carotid intima-media thickness (cIMT), C-reactive protein (CRP) and Young’s modulus, measures of arterial wall thickness, inflammation and arterial stiffness, respectively.

**Results:**

Levels of sRAGE and esRAGE correlated strongly both at baseline and at follow-up in both diabetes patients and controls. With increasing age, mean values of both variants declined, independent of gender, diabetes or pubertal stage. In the diabetes group, multiple regression analysis showed a positive association between both variants of soluble RAGE and cIMT. There was no significant relationship with Young’s modulus, but a negative association between sRAGE at baseline and CRP at follow-up. The ratios between the AGEs and the variants of soluble RAGE were increased in diabetes patients compared to controls.

**Conclusions:**

The results show a possible protective effect of high levels of sRAGE at baseline against inflammation 5 years later, but not on arterial stiffness or wall thickness, in this cohort of adolescents and young adults with T1D.

## Background

Advanced glycation end products (AGEs) have been implicated in the pathogenesis of both microvascular complications and accelerated atherosclerosis in type 1 diabetes (T1D) [1, 2]. A proposed mechanism for the deleterious effect of AGEs is engagement of the receptor for AGEs, RAGE, which is a multiligand transmembrane receptor in the immunoglobulin superfamily present on the surface of various cell types in atherosclerotic lesions [3–5]. Two of the AGEs that potentially bind to RAGE are carboxymethyllysine (CML) and methylglyoxal-derived hydroimidazolone-1 (MG-H1) [6, 7]. Receptor binding leads to activation of monocytes and the vascular endothelium and increases inflammation in the vessel wall [8, 9]. A mouse cell model indicates that RAGE also mediates AGE-induced macrophage migration [10].

RAGE has two soluble forms prevalent in human plasma. One created as the membrane bound receptor is cleaved by metalloproteinases [11], and the other produced by alternative splicing of the RAGE gene [12]. The latter is known as endogenous secretory RAGE (esRAGE) [13], and together they constitute plasma soluble RAGE (sRAGE). Estimates indicate that esRAGE accounts for 20–50 % of sRAGE [14, 15].

It has been postulated that both soluble variants of RAGE work as decoys for AGEs, reducing RAGE signaling and inflammation [16]. In a mouse model, treatment with intraperitoneal sRAGE suppressed the accelerated atherosclerosis of diabetes [17]. This suggests that high levels of sRAGE protect against the atherogenic effect of AGEs. Studies in T1D have, however, shown conflicting results. Levels of soluble variants of RAGE both lower [18–20] and higher [21–23] than controls have been demonstrated. Previously, Katakami et al. have found an inverse association between levels of esRAGE and carotid intima media thickness (cIMT) both cross-sectionally [18] and longitudinally in young adults [24]. Nin et al., however, showed a positive association between sRAGE and cardiovascular events and all-cause mortality [22, 25]. The seemingly contradictory results might be due to the different ethnic origin of the study populations [26], but more likely reflect the complexity of the regulation of the AGE-RAGE-sRAGE and AGE-RAGE-esRAGE axes. This implies that AGEs or sRAGE alone may not be particularly informative as biomarkers, and an AGE/sRAGE ratio has been proposed as an improvement [27].

In this study, we aimed to investigate longitudinally whether higher levels of soluble variants of RAGE protect against atherosclerosis development in young patients with T1D compared to healthy controls.

## Methods

### Study population

The atherosclerosis and childhood diabetes (ACD) prospective study was initiated in 2006, comprising 314 diabetes patients and 120 controls, all between 8 and 18 years old, from the South East health region of Norway. The non-diabetic controls were mainly classmates of the diabetes patients. The details of the cohort and inclusion process have previously been described [28]. From 2011 to 2013, the participants were encouraged to attend a 5-year follow-up, and 80 % agreed. An additional 38 control subjects were also enrolled at this time.

Both at baseline and at follow-up, blood samples were taken in the morning between 0800 and 1000 a.m. after an overnight fast. This was followed by carotid ultrasound and a clinical examination.

All parents and participants over 18 years old have given their written informed consent. The protocol, both at baseline and follow-up has been approved by the Norwegian Regional Committee for Research Ethics, and the study has been conducted according to the Declaration of Helsinki.

### Laboratory methods

All blood samples were centrifuged at 2500×*g* for 10 min immediately after venipuncture and stored at −80 °C until analysis. Serum sRAGE and esRAGE were measured using enzyme-linked immunosorbent assay (ELISA) kits (Quantikine, R&D Systems, Minneapolis, MN, USA and B-Bridge International Inc., Cupertino, CA, USA respectively) according to the manufacturer’s instructions. The inter-assay coefficients of variation (CV) in our laboratory were 5.8 % for sRAGE and 5.7 % for esRAGE. At baseline 411 participants (299 diabetes patients, 112 controls) had valid measurements. At follow-up 369 (241 diabetes patients and 128 controls) were tested. 318 participants (231 diabetes patients and 87 controls) had measurements at both baseline and follow-up. All samples from both timepoints were measured in the same run to avoid bias due to assay variability.

MG-H1 and CML were measured by dissociation-enhanced lanthanide fluorescent immunoassay (DELFIA) as previously described in detail [29–31].

Images of the common carotid arteries (CCAs) at baseline were obtained using a Siemens Acuson Sequoia 512 (Siemens Acuson; Mountain View, CA, USA) ultrasound scanner equipped with a linear array 14 MHz transducer. Further details have been described previously [28]. At follow-up, the images of the CCAs were obtained using a Zonare Z-one ULTRA (Zonare Medical Systems; Mountain View, CA, USA) ultrasound scanner equipped with a linear array 8 MHz transducer. The cIMT measurements were performed using M’Ath 3.2.0 (Intelligence in Medical Imaging; Paris, France). All cIMT measurements were from the far wall in end-diastole. Blood pressure for calculating Young’s modulus was assessed using a standard oscillometric device over the brachial artery.

CRP was determined by high sensitivity ELISA (DRG Instruments GmbH, Germany) with a detection limit of 0.1 mg/L. The inter-assay CV was <5 %.

HbA1c was measured at a DCCT-standardized laboratory using high performance liquid chromatography (Variant; Bio-Rad, Richmond, CA, USA), CV <3 %. Other routine laboratory analyses were performed by conventional methods.

### Statistical analysis

Demographic and clinical data are presented as either proportions, means with their standard deviations (SD) or medians with the 25th and 75th percentile. Differences in continuous variables between groups were tested with the Student *t* test, alternatively the Mann–Whitney *U*-test for non-normally distributed data. Correlation analyses between continous variables were performed using Pearson’s correlation coefficient (r) for normally distributed data, or otherwise Spearman’s rho (*ρ*). Univariate linear regression analysis was performed to identify associations between sRAGE and esRAGE as exposure variables and surrogate markers of atherosclerosis as outcome variables. Only variables with significant relationships with both the exposure and the outcome variables were considered as possible confounders and included in a multivariate analysis. Adjustment for multiple confounding factors was performed using multivariate linear regression analysis with a manual backward elimination procedure. CRP was log_e_-transformed to achieve normally distributed residuals. A significance level of 5 % was used.

In addition, multivariate linear regression analysis with a manual backward elimination procedure was performed to identify predictors for soluble variants of RAGE at follow-up. Any variable with a p value <0.20 from the univariate analysis was considered a candidate for the multivariate model. All statistical analyses were performed using the SPSS software package for Mac, version 19.0 (SPSS, Chicago, IL, USA).

## Results

Most of the diabetes patients (97 %) were on intensive insulin treatment using insulin pumps or >4 insulin injections a day. At baseline, comparison with the Norwegian Childhood Diabetes Registry showed that our cohort was a representative sample of the young T1D population in Norway regarding gender, stage of puberty, HbA1c, blood pressure and lipid status [28]. Due to exclusion of participants under 8 years of age, the patients in our cohort were slightly older, had longer diabetes duration, higher body mass index (BMI) and were more frequently pump users than the rest of the Norwegian children and adolescents with diabetes. The clinical and metabolic characteristics of the patients at baseline and follow-up are shown in Table 1. The diabetic patients had higher body weight, blood pressure and increased serum lipid levels compared to the control subjects both at baseline and at follow-up.

**Table 1:** Clinical and metabolic characteristics

	Baseline			5 year follow-up		
	Diabetes (n = 299)	Controls (n = 112)	p value	Diabetes (n = 241)	Controls (n = 128)	p value
Diabetes duration (years)^a^	5.1 (2.9, 7.7)			9.6 (7.5, 12.6)		
Insulin pump users (%)	53.3			61.3		
Age (years)	13.7 (2.8)	13.4 (2.5)	0.207	18.7 (2.8)	18.7 (2.9)	0.895
Girls, n (%)	150 (50.2)	64 (57.1)	0.224	129 (53.5)	72 (56.3)	0.661
Height (cm)	160.5 (14.4)	157.6 (13.3)	0.060	171.2 (9.1)	172.1 (9.2)	0.383
Weight (kg)	54.8 (16.7)	48.5 (13.2)	<0.001	71.0 (14.6)	66.9 (12.5)	0.006
BMI (kg/m^2^)	20.8 (3.9)	19.2 (3.1)	<0.001	24.1 (4.3)	22.5 (3.4)	<0.001
Waist circumference (cm)	71.1 (10.0)	66.8 (6.6)	<0.001	79.0 (9.6)	75.5 (8.2)	<0.001
Systolic blood pressure (mmHg)	101 (10.1)	98 (10)	0.025	112 (11)	111 (10)	0.656
Diastolic blood pressure (mmHg)	60 (8)	58 (7)	0.017	70 (8)	68 (8)	0.024
HbA1c (%) [mmol/mol, SD]	8.4 (1.2) [68, 13.1]	5.3 (0.3) [34, 3.3]	<0.001	9.0 (1.4) [75, 15.3]	5.2 (0.3) [33, 3.3]	<0.001
Total cholesterol (mmol/L)	4.6 (0.8)	4.3 (0.7)	0.001	4.8 (1.0)	4.4 (1.0)	0.001
HDL (mmol/L)	1.8 (0.4)	1.7 (0.4)	0.048	1.6 (0.5)	1.6 (0.4)	0.161
LDL (mmol/L)	2.5 (0.7)	2.3 (0.7)	0.016	2.7 (0.8)	2.5 (0.8)	0.014
Triglycerides (mmol/L)^a^	0.7 (0.5, 0.9)	0.7 (0.5, 0.9)	0.367	0.9 (0.6, 1.3)	0.8 (0.6, 1.0)	0.024
Apolipoprotein B (g/L)	0.74 (0.19)	0.66 (0.17)	<0.001	0.90 (0.26)	0.79 (0.23)	<0.001
Apolipoprotein A1 (g/L)	1.55 (0.28)	1.44 (0.33)	0.001	1.56 (0.31)	1.48 (0.27)	0.006
ApoB/ApoA1	0.5 (0.2)	0.5 (0.3)	0.698	0.6 (0.2)	0.5 (0.2)	0.025
Urine albumin/creatinine (mg/mmol)^a^	0.70 (0.40, 1.30)	0.63 (0.36, 1.33)	0.688	0.65 (0.30, 1.55)	0.34 (0.19, 0.90)	0.001

Mean values (SD)

^a^Median (25th and 75th percentile)

The results from the sRAGE and esRAGE assays and the measurements of surrogate markers of atherosclerosis are presented in Table 2. We found no difference in soluble variants of RAGE between the groups, except for lower levels of esRAGE in the diabetes group at baseline. As for cIMT and Young’s modulus, we found no significant differences between the groups. CRP was significantly elevated in the diabetes group both at baseline [32] and at follow-up.

**Table 2:** Soluble RAGE and surrogate markers of atherosclerosis

	Baseline			5 year follow-up		
	Diabetes	Controls	p value	Diabetes	Controls	p value
sRAGE (pg/ml)	1664 (602)	1773 (574)	0.100	1578 (589)	1585 (561)	0.909
esRAGE (ng/ml)	0.36 (0.16)	0.42 (0.16)	<0.001	0.33 (0.21)	0.33 (0.13)	0.810
cIMT (mm)	0.45 (0.05)	0.44 (0.04)	0.067	0.51 (0.05)	0.51 (0.06)	0.324
Young’s modulus (kPa)	13.7 (2.8)	13.4 (2.5)	0.207	18.7 (2.8)	18.7 (2.9)	0.895
CRP (mg/l)^a^	0.50 (0.27, 1.84)	0.31 (0.19, 0.67)	<0.001	1.16 (0.43, 3.64)	0.69 (0.28, 2.00)	0.002

Mean values (SD)

^a^Median (25th and 75th percentile)

The levels of sRAGE and esRAGE correlated strongly, both in the diabetes and the control group (Table 3). In the diabetes group, the boys had significantly higher sRAGE both at baseline [1752 (SD = 609) vs. 1576 (SD = 584) pg/ml, p = 0.011] and at follow-up [1682 (SD = 609) vs. 1487 (SD = 557) pg/ml, p = 0.010] as well as higher esRAGE at baseline [0.39 (SD = 0.17) vs. 0.34 (SD = 0.15) ng/ml, p = 0.005] than the girls. These differences disappeared when excluding the girls using oral contraceptives from the analysis.

**Table 3:** Coefficients of correlation (Pearson), all with p < 0.001

	Diabetes group			Control group		
	esRAGE baseline	esRAGE follow-up	sRAGE baseline	esRAGE baseline	esRAGE follow-up	sRAGE baseline
esRAGE follow-up	0.396	1		0.636	1	
sRAGE baseline	0.800	0.295	1	0.775	0.597	1
sRAGE follow-up	0.593	0.510	0.638	0.458	0.828	0.620

At baseline, there was a weak negative correlation between HbA1c and sRAGE (r = −0.148, p = 0.011) and esRAGE (r = −0.137, p = 0.018). There were no significant correlations with HbA1c at follow-up or in the control group at any time point.

### Natural progression of sRAGE and esRAGE

**Fig. 1:**
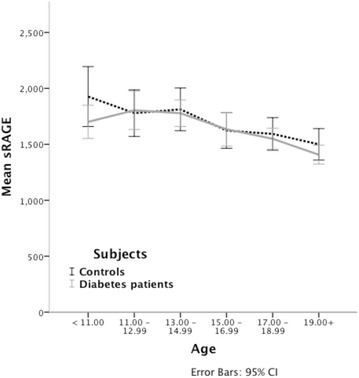
Mean sRAGE and age. Values in diabetes patients (*grey line)* and controls (*dotted line*). The figure includes two values from each participant, one at baseline and one at follow-up

There was a decline in sRAGE and esRAGE levels with increasing age. Figure 1 shows levels of sRAGE in diabetes patients and controls. For illustrative purposes, values from each patient at both baseline and follow-up are included. The graphs for esRAGE as well as for both soluble variants of RAGE when separated by gender showed similar patterns. There was no decline in the levels of either soluble variant of RAGE across the five Tanner stages of puberty.

To determine whether this relationship with age was confounded by other factors, we performed regression analyses. At baseline, the association between age and esRAGE remained significant after controlling for the confounding effect of CRP in the diabetes group (B = −0.009, R^2^ = 0.082 and p = 0.009) and in girls with diabetes (B = −0.012, R^2^ = 0.123 and p = 0.008). Also, sRAGE was significantly associated with age in the control group (B = −47.1, R^2^ = 0.081 and p = 0.033, controlling for MG-H1), in girls with diabetes (B = −61.6, R^2^ = 0.154 and p = 0.001, controlling for CRP) and in control girls (B = −99.1, R^2^ = 0.154 and p = 0.001, no confounding variables). At follow-up, esRAGE was not associated with age in any group. There was, however, significant associations between sRAGE and age in the diabetes group (B = −51.5, R^2^ = 0.131 and p < 0.001, controlling for CRP), in boys with diabetes (B = −57.8, R^2^ = 0.071 and p = 0.004, no confounding variables) and in girls with diabetes (B = −45.5, R^2^ = 0.152 and p = 0.016, controlling for CRP). When identifying predictors of esRAGE or sRAGE in the different groups, age was only significant for sRAGE at follow-up in the diabetes group.

### Soluble variants of RAGE and atherosclerosis

In multiple regression analyses we found significant associations between soluble variants of RAGE and the surrogate markers of atherosclerosis cIMT and CRP in the diabetes group. These are presented in Table 4. There were no significant associations in the control group, and there were no significant associations involving Young’s modulus in either group.

**Table 4:** Significant associations in the diabetes group

Independent variable	Dependent variable	Beta value	SE	p value	R^2^	Confounding variables
Baseline esRAGE	Follow-up cIMT	0.074	0.023	0.001	0.141	Gender, waist circumference, BMI
Baseline sRAGE	Follow-up cIMT	1.4 × 10^−5^	6.2 × 10^−6^	0.027	0.116	Gender, waist circumference, ApoB
Baseline sRAGE	Follow-up CRP	−3.3 × 10^−4^	1.4 × 10^−4^	0.017	0.269	Height, medication, BMI

We also analyzed delta values (follow-up minus baseline). We found no significant associations between baseline variants of soluble RAGE and delta CRP in either patient group. Due to different measurement techniques applied at each time point, we were unable to evaluate delta values for cIMT and Young’s modulus. Delta sRAGE and delta esRAGE were not correlated with either delta HbA1c, delta LDL or delta BMI.

### AGE/soluble variants of RAGE ratios

At baseline, levels of MG-H1 were significantly increased in the diabetes group compared to controls, 154.4 (SD = 40.8) vs. 142.8 (SD = 35.5) U/ml, p = 0.008. There was no significant difference in CML, 18.0 (SD = 4.4) vs. 17.3 (SD = 5.7) U/ml, p = 0.152. All possible AGE/soluble variants of RAGE ratios at baseline were significantly increased in the diabetes patients compared to controls (Table 5). In multivariate analyses, there were no significant associations between AGE/soluble variants of RAGE ratios at baseline and surrogate markers of atherosclerosis 5 years later.

**Table 5:** AGE/soluble RAGE ratios

Ratio	Diabetes patients	Controls	p value
MG-H1/sRAGE	0.099 (0.071, 0.124)	0.083 (0.058, 0.117)	0.006
MG-H1/esRAGE	461 (335,624)	332 (248,480)	<0.001
CML/sRAGE	0.012 (0.008, 0.015)	0.010 (0.007, 0.013)	0.005
CML/esRAGE	53.4 (39.8, 72.4)	38.1 (28.6, 58.6)	<0.001

Median (25th, 75th percentile)

## Discussion

The main findings in this study are the relationships between the soluble variants of RAGE and surrogate markers of atherosclerosis. Our analyses do not indicate a protective effect of high levels of sRAGE or esRAGE against atherosclerosis development measured by cIMT. In fact, in the diabetes group we found the opposite, that high levels of both sRAGE and esRAGE at baseline are significantly associated with higher cIMT 5 years later. This is in line with the studies by Nin et al. [22, 25]. On the other hand, baseline sRAGE was significantly negatively associated with CRP, indicating a protective effect against inflammation. We found no significant associations between soluble variants of RAGE and any measure of atherosclerosis in the control group.

We have demonstrated a reduction in values of both sRAGE and esRAGE in the teens and early twenties, regardless of diabetes, gender, or pubertal stage. The association with age remained significant in several groups when controlling for confounding factors, particularly in girls. A similar reduction with increasing age has been shown in women below the age of 35 compared to older subjects [33]. Komosinska-Vassev et al. have also shown decreasing esRAGE values with increasing age in subjects without significant disease, particularly in women [34]. The participants in our study were young and healthy, apart from T1D. The fact that we did not find an association between T1D and the reduction in sRAGE or esRAGE values suggests that this is a normal physiologic development. Most studies, however, show a significant relationship between soluble variants of RAGE and disease states [35]. High levels of sRAGE are associated with healthy aging [36], but whether sRAGE is protective against disease, reduced in disease, or both, is unknown.

The strong correlation between baseline and follow-up values of both sRAGE and esRAGE indicate that changes in levels of soluble variants of RAGE over time in each patient are generally small. We also found strong correlations between sRAGE and esRAGE both at baseline and follow-up. This suggests that esRAGE constitutes a large proportion of the measured sRAGE and consequently that the cleaved form of RAGE is less prevalent. This is in contrast to most other studies, where esRAGE is estimated to constitute only 20 % of sRAGE [11, 14, 37, 38]. A possible explanation is that our subjects are younger and healthier than in the previous studies. This is supported by the only study that has assessed the levels of sRAGE and esRAGE using comparable immunoassays [15]. Higher levels of esRAGE compared to sRAGE in youth is reasonable since RAGE amplifies inflammation [9]. Older patients with more pronounced atherosclerosis or other inflammatory diseases would express more RAGE on the cell surface and subsequently release more cleaved RAGE into the circulation [39].

The regulation of the AGE-RAGE-sRAGE and AGE-RAGE-esRAGE axes is only partly understood. RAGE is subject to regulated intramembrane proteolysis by ADAM10 (a disintegrin and metalloproteinase 10), shedding the cleaved variant of sRAGE in a process dependent on intracellular calcium levels [40]. Shedding can also be induced by activation of G protein-coupled receptors [41]. As for esRAGE, engagement of RAGE by methylglyoxal-modified human serum albumin stimulated production of RAGE and esRAGE to a similar extent in human umbilical vein endothelial cells. Interestingly, engagement of RAGE by carboxymethyllysine-modified human serum albumin more selectively stimulated production of RAGE [42]. Ohe et al. have shown that elements in exon 9B of the RAGE gene can modulate the ratio of RAGE/esRAGE production [43]. Levels of sRAGE are associated with albuminuria, indicating that kidney function is important for esRAGE excretion [38]. Still, much is yet left to discover about the production and excretion of the soluble variants of RAGE. Even though sRAGE in our study was negatively associated with CRP 5 years later, this does not necessarily imply a causal relationship. The finding indicates a protective effect, but as inflammation most likely alters the AGE-RAGE-sRAGE and AGE-RAGE-esRAGE axes in a still unpredictable fashion, sound conclusions are difficult to reach.

The AGE/sRAGE ratio has been proposed as an improved biomarker for the AGE-RAGE-sRAGE axis [27]. A higher ratio is associated with pre-eclampsia in women with T1D [44], and we also found higher ratios in diabetes patients compared to controls in this study. However, we found no significant associations with surrogate markers of atherosclerosis, rendering the ratios less useful than sRAGE alone. Furthermore, the ratios are hardly easier to interpret than the separate factors, as the influence of other AGEs, membrane bound RAGE, production and excretion of sRAGE and esRAGE are still unknown.

### Strengths and limitations

The strengths of this study include the prospective design, relatively large cohort of diabetes patients and controls and different surrogate markers of atherosclerosis. There are of course also limitations. Non-invasive measurement of atherosclerosis in children and young adults have several inherent shortcomings. Only cIMT and CRP are accepted surrogate markers of atherosclerosis, and best documented in adults [45]. Young’s modulus is not the gold standard for measuring arterial stiffness, which introduces some uncertainty. It is debatable whether AGE’s in the concentration found in vivo actually are important ligands for RAGE, and there are several other classes of ligands for RAGE not considered in this study, including the proteins S100/calgranulin and high mobility group box 1 (HMGB1) and others [46, 47]. As long as the AGE-RAGE-sRAGE and AGE-RAGE-esRAGE axes are insufficiently understood, the data must be interpreted with caution.

## Conclusions

In this study, we have shown a possible protective effect of high levels of sRAGE on inflammation, but not on arterial stiffness or wall thickness, in this cohort of adolescents and young adults with T1D. We have also shown an age-associated decline in values of both sRAGE and esRAGE in the teens and early twenties of both T1D patients and healthy control subjects.

## Abbreviations

AGE: advanced glycation end product
RAGE: receptor for AGEs
esRAGE: endogenous secretory RAGE
sRAGE: soluble RAGE
MG-H1: methylglyoxal-derived hydroimidazolone-1
CML: carboxymethyllysine
cIMT: carotid intima-media thickness
CRP: C-reactive protein
T1D: type 1 diabetes
ACD: atherosclerosis and childhood diabetes
ELISA: enzyme-linked immunosorbent assay
CV: coefficient of variation
DELFIA: dissociation-enhanced lanthanide fluorescent immunoassay
CCA: common carotid artery
SD: standard deviation
ADAM10: a disintegrin and metalloproteinase 10
HMGB1: high mobility group box 1
